# Cerebral Salt Wasting Syndrome Caused by Severe Traumatic Brain Injury in a Pediatric Patient and Review of the Literature

**DOI:** 10.1155/2021/6679279

**Published:** 2021-10-22

**Authors:** Mohamed Aziz Daghmouri, Maroua Ouesleti, Mohamed Amine Touati, Olfa Faten, Sameh Zakhama, Lotfi Rebai

**Affiliations:** Department of Anesthesia, Trauma Center of Ben Arrous, Tunisia

## Abstract

**Background:**

Following acute traumatic brain injury, cerebral salt wasting (CSW) syndrome is considered as an important cause of hyponatremia apart from syndrome of inappropriate antidiuretic hormone. Differentiation between the two syndromes is crucial for the initiation of an adequate treatment. *Case Presentation.* We report a 15-year-old female adolescent, admitted to intensive care for acute severe traumatic brain injury. During his hospitalization, she developed a hyponatremia with an increase of urine output and hypovolemia. So, the most probable diagnosis was CSW. Initially, she was treated by hypertonic saline and volume expansion. However, his sodium level continued to fall despite infusion of hypertonic saline. That is why fludrocortisone was introduced initially at 50 *μ*g/day then increased to 150 *μ*g/day. Fludrocortisone was continued for the next months. Serum sodium level was 138 mmol/L after one month of treatment.

**Conclusion:**

Hyponatremia may occur after severe traumatic brain injury that is why an adequate treatment initiated on time is necessary in order to reduce morbidity and mortality.

## 1. Introduction

Cerebral salt wasting (CSW) syndrome is an uncommon cause of hyponatremia in neurosurgical patients especially following traumatic brain injury. Distinguishing it from the more familiar syndrome of inappropriate antidiuretic hormone (SIADH) is crucial, and it is vital to make rapidly the right diagnosis in order to start an appropriate treatment based on volume resuscitation and sodium restoration. Although CSW has rarely been studied in the traumatic brain injury population especially pediatric ones that is why we report a 15-year-old female child with acute brain injury complicated of CSW managed by saline hydration and fludrocortisone.

Written informed consent was obtained from the patient family for publication of this case report and accompanying images. This manuscript adheres to the SCARE guidelines.

## 2. Case Presentation

A 15-year-old female child was brought to the emergency department after a motor vehicle accident (pedestrian struck by motor vehicle). She had no significant past medical history and was not taking any regular medications. Initial physical examination revealed neurological distress with Glasgow come scale score of 4, decerebration, and bilateral mydriasis. Pulse rate was 100/min, blood pressure 120/65 mmHg, respiratory rate 30/min, and oxygen saturation 95%. Rapidly, she was intubated and received osmotherapy with hypertonic saline.

Computed tomography revealed left fronto-temporal subdural hematoma (6 mm) with left temporal commitment and diffuse cerebral edema. Neurosurgical opinion was sought. Therefore, she was admitted to intensive care unit. Initial complete blood cell count and serum biochemistry were without abnormalities, and serum sodium concentration was 140 mmol/L.

On day 6 of admission, the patient presented a significant increase of urine output (more than 3 mL/kg/h) with abnormalities in the transcranial Doppler ultrasonography. It was due to a low sodium level (124 mmol/L). Possibility of cerebral salt wasting (CSW) and syndrome of inappropriate antidiuretic hormone (SIADH) was considered. Urine osmolality and urine sodium were 390 mosmol/L and 114 mmol/L, respectively. Fractional excretion of potassium was higher (FEK > 15%). She was also hypovolemic so referred to the endocrinologist and nephrologist opinion; CSW was the most suitable diagnostic.

Correction was started rapidly using hypertonic saline (2% saline) and substantial volume replacement (equivalent of more than 3 L/day of 2% saline) in order to restore serum sodium to low normal levels within 48 h (sodium 135 mmol/L). Saline infusion (1.2% saline) was given as maintenance fluid during his hospitalization. However, on day 12 of admission, his sodium level continued to fall despite infusion of hypertonic saline. That is why fludrocortisone was introduced initially at 50 *μ*g/day then increased to 150 *μ*g/day. Introduction of this molecule resulted in a fall in requirements for hypertonic saline. Although, starting from day 25 of admission, serum sodium levels remained stable around 135 mmol/L on fludrocortisone alone, and she was discharged home 30 days postinjury.

It is worth noting that during his hospitalization, magnetic resonance imaging of the brain was done objectifying an encephalitis treated by antibiotics (linezolid and meropenem).

Fludrocortisone was continued for the next months. Serum sodium level was 138 mmol/L after one month of treatment. Since she was discharged; she has been followed by an endocrinologist.

## 3. Discussion

Cerebral salt wasting is resulting in hyponatremia and poorly hypovolemia caused by renal loss of sodium following intracranial disorders [1] which was first described by Peters et al. [2] in 1950. The mechanisms leading to CSW remain an area of debate, and one of the hypothesis is that, after a brain injury, there is an increasing of level natriuretic peptide hormones which inhibit sodium reabsorption and decrease release of renin [3], although CSW is associated with concentrated urines and high fractional excretion of urate and those parameters could help in order to distinguish CSW from other syndromes [4].

It is important and complex to distinguish CSW from the syndrome of inappropriate secretion of antidiuretic of hormone (SIADH) as treatments are different. The combination of excess fluid and hyponatremia in SIADH is treated by water restriction whereas in CSW hypovolemia necessitates replacement of both water and sodium. It is crucial to treat hyponatremia correctly because it can worsen cerebral edema or result in seizure; however, when it is suboptimally treated, it would cause osmotic demyelination [5]. In addition, one of proposed treatment of SIADH is Vaptan which is selective vasopressin receptor antagonists, and it is usually used when water restriction is insufficient [6].

CSW is most commonly studies in patients with aneurysmal subarachnoid hemorrhage with high incidence of hyponatremia up to 57% according to Sherlock et al. [7]. Although traumatic brain injury (TBI) could be associated with hyponatremia, however, literature studying CSW in TBI population is poor with little information available on physiopathology and outcomes. Furthermore, Leonard et al. [8] found that incidence of CSW in TBI patients was from 0.8% to 34.6%, and it was developed within days to two months postinjury. It occurs in patients of all ages, but it has been suggested that CSW takes a different course in children compared to adults. Nine pediatric cases of CSW due to TBI were published in the literature as well as we know (Table 1). Three patients developed CSW two days posttraumatic [9–11], two developed CSW after one week [10, 12], and four developed CSW between two weeks and two months posttraumatic [13–16].

Concerning the management of CSW, it is based on the correction of intravascular volume depletion and hyponatremia. However, close monitoring of saline sodium level is crucial in order to prevent overly rapid correction of hyponatremia [17]. Moreover, pharmacological intervention could be necessary in some cases especially fludrocortisone which is recommended as a potential therapeutic option. Misra et al. [18] proved that fludrocortisone may result in earlier normalization of serum sodium in patients with cerebral salt wasting as a part of tuberculous meningitis.

## 4. Conclusion

Hyponatremia could be present after traumatic brain injury. Every clinician should be aware of the importance of making the right diagnosis on time and distinguishing CSW from SIADH which they have opposite treatment. So, we reported this case in order to emphasize the role of initiating appropriate treatment rapidly in reducing morbidity and mortality of hyponatremia.

## Figures and Tables

**Table 1:** Summary of pediatric case reports examining cerebral salt wasting after traumatic brain injury.

Author (years)	Case	Head CT–MRI	Timing to development of CSW	Treatment	Outcomes
(1) Chaudhary [13] (2016)	17-month-old female: closed head trauma, GCS 10/15	Subdural hematoma, subarachnoid hemorrhage, extradural hematoma, and contusion	10 days	Saline hydration and fludrocortisone 200 *μ*g/day	Improved
(2) Simsek [14] (2008)	6-month-old female: closed head and cervicothoracic trauma	8 mm benign congenital subdural collection over frontotemporal lobes	1 month	Saline hydration	Improved
(3) Askar [15] (2007)	17-year-old male: closed head trauma and multiple injuries to the face, chest, and pelvis due to MVA	—	15 days	Saline hydration and fludrocortisone 300 *μ*g/day	Improved
(4) Steelman [9] (2008)	9-year-old male: laceration to the chin and closed head trauma	—	2 days	Saline hydration	Improved
(5) Berkenbosch [10] (2002)	15-year-old male: severe closed head injury from cycling accident	Right-sided frontal contusion	2 days	Saline hydration	Improved
(6) Berkenbosch [10] (2002)	6-year-old male: severe closed head injury	1.5 cm left frontoparietal contusion, marked diffuse cerebral edema	6 days	Saline hydration	Improved
(7) Donati-Genet [12] (2001)	4-year-old male: closed head injury, multiple bone fractures, chest trauma	Day 5 CT after seizure: diffuse cerebral edema and small cerebellar hemorrhage	5 days	Saline hydration	Improved
(8) Kappy [16] (1996)	6-month-old male: MVA with normal initial evaluation. Over next 2 months, vomiting and increasing head circumference	2 months postaccident bilateral subdural fluid accumulation	2 months	Saline hydration	Improved
(9) Ganong [11] (1993)	5-year-old male: closed head injury due to MVA	—	2 days	Saline hydration	Improved

CSW: cerebral salt wasting; CT: computer tomography; MRI: magnetic resonance imaging; GCS: Glasgow coma scale; MVA: motor vehicle accident.

## References

[1] Betjes M. G., Koopmans R. P. (2000). Hyponatremia in acute intracranial disorders: cerebral salt wasting. *Nederlands tijdschrift voor geneeskunde*.

[2] Peters J. P., Welt L. G., Sims E. A. H., Orloff J., Needham J. (1950). A salt-wasting syndrome associated with cerebral disease. *Transactions of the Association of American Physicians*.

[3] Yee A. H., Burns J. D., Wijdicks E. F. M. (2010). Cerebral salt wasting: pathophysiology, diagnosis, and treatment. *Neurosurgery Clinics of North America*.

[4] Maesaka J. K., Imbriano L., Mattana J., Gallagher D., Bade N., Sharif S. (2014). Differentiating SIADH from cerebral/renal salt wasting: failure of the volume approach and need for a new approach to hyponatremia. *Journal of Clinical Medicine*.

[5] Tam C. W., Shum H. P., Yan W. W. (2019). Impact of dysnatremia and dyskalemia on prognosis in patients with aneurysmal subarachnoid hemorrhage: a retrospective study. *Indian journal of critical care medicine*.

[6] Büttner S., Bachmann J., Geiger H., Obermüller N. (2017). Long-term vaptan treatment of idiopathic SIADH in an octogenarian. *Journal of clinical medicine*.

[7] Sherlock M., O'Sullivan E., Agha A. (2009). Incidence and pathophysiology of severe hyponatraemia in neurosurgical patients. *Postgraduate Medical Journal*.

[8] Leonard J., Garrett R. E., Salottolo K. (2015). Cerebral salt wasting after traumatic brain injury: a review of the literature. *Scandinavian journal of trauma, resuscitation and emergency medicine*.

[9] Steelman R., Corbitt B., Pate M. F. D. (2006). Early onset of cerebral salt wasting in a patient with head and facial injuries. *Journal of Oral and Maxillofacial Surgery*.

[10] Berkenbosch J. W., Lentz C. W., Jimenez D. F., Tobias J. D. (2002). Cerebral salt wasting syndrome following brain injury in three pediatric patients: suggestions for rapid diagnosis and therapy. *Pediatric Neurosurgery*.

[11] Ganong C. A., Kappy M. S. (1993). Cerebral salt wasting in children. The need for recognition and treatment. *American Journal of Diseases of Children*.

[12] Donati-Genet P. C., Dubuis J. M., Girardin E., Rimensberger P. C. (2001). Acute symptomatic hyponatremia and cerebral salt wasting after head injury: an important clinical entity. *Journal of Pediatric Surgery*.

[13] Chaudhary N., Pathak S., Gupta M. M., Agrawal N. (2016). Cerebral salt wasting syndrome following head injury in a child managed successfully with fludrocortisone. *Case Reports in Pediatrics*.

[14] Simsek E., Dilli D., Yasitli U., Ozlem N., Bostanci I., Dallar Y. (2008). Cerebral salt wasting in a child with cervicothoracic hematoma. *Journal of Pediatric Endocrinology & Metabolism*.

[15] Askar A., Tarif N. (2007). Cerebral salt wasting in a patient with head trauma: management with saline hydration and fludrocortisone. *Saudi Journal of Kidney Diseases and Transplantation*.

[16] Kappy M. S., Ganong C. A. (1996). Cerebral salt wasting in children: the role of atrial natriuretic hormone. *Advances in Pediatrics*.

[17] Taylor P., Dehbozorgi S., Tabasum A. (2017). Cerebral salt wasting following traumatic brain injury. *Endocrinology, Diabetes & Metabolism Case Reports*.

[18] Misra U. K., Kalita J., Kumar M. (2018). Safety and efficacy of fludrocortisone in the treatment of cerebral salt wasting in patients with tuberculous meningitis: a randomized clinical trial. *JAMA Neurology*.

